# Acute Hypocalcemia and Metabolic Alkalosis in Children on Cation-Exchange Resin Therapy

**DOI:** 10.1155/2017/6582613

**Published:** 2017-08-01

**Authors:** Aadil Kakajiwala, Kevin T. Barton, Elisha Rampolla, Christine Breen, Madhura Pradhan

**Affiliations:** ^1^Division of Pediatric Nephrology, Washington University in St. Louis School of Medicine, St. Louis, MO, USA; ^2^Divisions of Nephrology, The Children's Hospital of Philadelphia, Philadelphia, PA, USA; ^3^Perelman School of Medicine, The University of Pennsylvania, Philadelphia, PA, USA

## Abstract

**Background:**

Sodium polystyrene sulfonate (SPS) is a chelating agent used for the treatment of hyperkalemia. SPS has a wide range of exchange capacity requiring close monitoring of serum electrolytes. We observed two patients who developed acute hypocalcemia and increased metabolic alkalosis after initiating SPS therapy. We report these cases to draw attention to the potential risk of this medication in pediatric patients.

**Case Diagnosis/Treatment:**

Two children with chronic kidney disease on dialysis were started on SPS for hyperkalemia. Within a week after initiation of the medication, both patients developed hypocalcemia on routine labs without overt clinical manifestations. The hypocalcemia was rapidly corrected with oral supplementation and discontinuation of SPS.

**Conclusions:**

Severe hypocalcemia can develop after SPS therapy. The metabolic alkalosis in these patients associated with the hypocalcemia put them at increased risk for complications. Hence, careful attention must be paid to the state of calcium metabolism in all patients receiving SPS. Often calcium supplementation is required to maintain normal calcium levels.

## 1. Introduction

Sodium polystyrene sulfonate (SPS) is a cross-linked polymer, used in the treatment of hyperkalemia. It contains sulfonic groups bound to sodium ions that are easily exchanged for other cations in solution. When taken orally or rectally, the resin exchanges sodium ions for potassium ions in the intestine before being excreted. SPS may also be added to formula as a chelating agent to decrease potassium load.

SPS has such a wide range of exchange capacity that close monitoring of serum electrolytes is necessary. Its principle side effect has been excessive sodium load, but hypokalemia, hypocalcemia, and even tetany have been described after SPS therapy [1, 2]. We report two patients who developed acute hypocalcemia and increased metabolic alkalosis after initiating SPS therapy. These cases draw attention to the potential risk of this medication in pediatric patients.

## 2. Case Presentation

### 2.1. Case  1

An 18-month-old male child with steroid resistant nephrotic syndrome due to diffuse mesangial sclerosis rapidly progressed to end stage renal disease. He was initially started on hemodialysis followed by chronic peritoneal dialysis (PD). His chronic medications included lansoprazole, hydrocortisone, sevelamer, levothyroxine, amlodipine, epogen, ferrous sulfate, multivitamin, erythromycin, and albuterol. His main source of nutrition was a peptide-based formula that provided 1008 mg of calcium, 540 IU of vitamin D, and 30.4 mEq of potassium per day. The dialysate contained 2.5 mEq/l of calcium.

**Figure 1:**
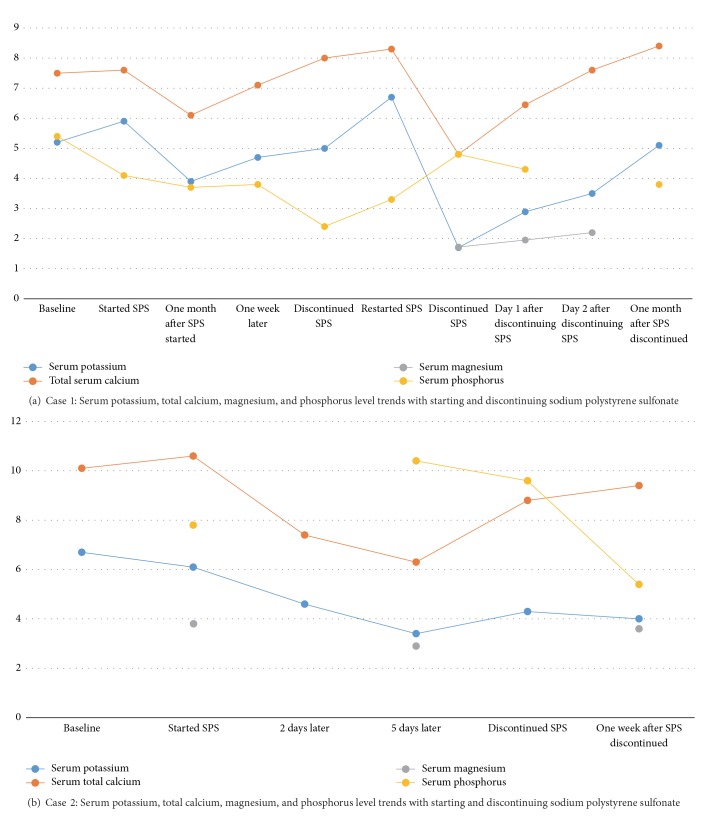


A year after being on PD, he developed persistent hyperkalemia (serum potassium 5.2 to 5.9 mEq/L) with increased doses of enalapril and was started on 0.5 g/kg of SPS. A month later, routine blood work noted improved serum potassium at 3.9 mEq/l, metabolic alkalosis (serum bicarbonate of 35 mEq/l), hypocalcemia (calcium of 6.1 mg/dl, serum albumin of 3.3 g/dl), and phosphorus of 3.7 mg/dL (Figure 1(a)). Intact PTH level had increased in the last month from 118.4 pg/ml to 180.4 pg/ml and 25(OH)-Vitamin D checked 6 months earlier was 33 ng/dL. On examination, heart rate was about 130/min and blood pressure was 102/58 mmHg. He had no tetany, normal deep tendon reflexes, a negative Chvostek sign, and no electrocardiogram (EKG) changes. He was started on 0.1 mcg of calcitriol weekly and 350 mg of calcium carbonate supplement three times a day (administered in between feeds). In a week his potassium was 4.7 mEq/L and calcium improved to 7.1 mg/dL. The following month, he had a potassium of 5 mEq/L, serum bicarbonate of 36 mEq/L, serum calcium concentration of 8 mg/dl (albumin of 3.7 g/dL), phosphorus of 2.4 mg/dl, and intact PTH level of 190 pg/ml. He had blood pressure of about 90/60 mmHg and enalapril was stopped. Sevelamer was decreased from 4.8 g to 3.2 g and SPS was discontinued.

A week later, he was noted to have a serum potassium concentration of 6.7 mEq/L, serum bicarbonate of 30 mEq/L, calcium of 8.3 mg/dl, and phosphorus of 3.3 mg/dL. He was started on 30 g of SPS (0.8 g of SPS per mEq of potassium in the formula) added to his formula for decanting. He was continued on calcium carbonate three times a day (administered with bolus feeds) and sevelamer was discontinued. Repeat serum potassium concentration was 5 mEq/L.

He had episodes of vomiting and diarrhea while being on SPS. About one week later, he had routine blood work done which was again notable for hypokalemia (serum potassium concentration of 1.7 mEq/L), persistent metabolic alkalosis (serum bicarbonate 35 mEq/L), and severe hypocalcemia (serum calcium of 4.8 mg/dL, albumin level of 3.3 g/dL). The serum phosphorus level was 4.8 mg/dL and magnesium 1.72 mg/dL. In the ED, he had a transient breath holding spell, heart rate of about 120/min and blood pressure of 114/55 mmHg, no tetany, normal deep tendon reflexes, and a normal EKG. He was treated with intravenous calcium and potassium. SPS was discontinued, calcium supplements were increased to 810 mg/day, and he was dialyzed on high calcium (3.5 mEq/L) dialysate fluid with 1 mEq/L of potassium. At the time of discharge his serum potassium concentration was 4.3 mEq/L, calcium concentration was 7.9 mg/dL, serum phosphorus level was 4 mg/dL, and magnesium level was 2.2 mg/dL. Most recently, he has maintained a stable calcium of about 9.4 mg/dL while off of SPS.

### 2.2. Case  2

Second case is male with chronic kidney disease (CKD) secondary to posterior urethral valves who was maintained on chronic PD since 8 months of age.

At 17 months of age, while on PD, his serum potassium and phosphorus were low at 3.0 mEq/L and 2.9 mg/dL, respectively, for which he was started on 15 mEq of potassium phosphate supplements per day. He was not receiving SPS at that time. Two weeks later, he developed stridor with agitation and was diagnosed with croup. He improved after treatment with racemic epinephrine and dexamethasone. The following week, his symptoms returned and he again was diagnosed with croup. His laboratory tests were significant for severe hypocalcemia (serum calcium of 4.2 mg/dL and ionized calcium < 0.25 mmol/L). This episode was attributed to recent initiation of potassium phosphate. Hypocalcemia resolved with intravenous and enteral calcium supplementation and discontinuation of potassium phosphate.

At 28 months of age, his chronic medications included cholecalciferol (400 IU daily), lansoprazole, 2.5 mEq daily potassium chloride, somatotropin, metoclopramide, Nephronex, ferrous sulfate, and epogen. He was on formula feeds with Suplena (given by mouth and G-tube) that contained 570 mg of calcium, 45 IU of vitamin D daily, and 15.5 mEq of potassium. The dialysate contained 2.5 mEq/L of calcium. His serum bicarbonate level was 26 mEq/l, serum calcium was 10.1 mg/dl (albumin concentration of 3.9 g/dl), phosphorus was 7.3 mg/dl, intact PTH concentration was 801 pg/ml, and 25(OH)-Vitamin D concentration was 67.2 ng/ml. The cholecalciferol was discontinued. Sevelamer (1600 mg added to the formula for decanting) was started at this visit. About one week later he underwent lysis of intra-abdominal adhesions due to pain and abdominal distention with PD. Due to recurrence of symptoms and leakage of PD fluid, he was admitted for initiation of hemodialysis (HD) due to ultrafiltration failure.

At the time of initiation of HD he had a serum bicarbonate concentration of 31 mEq/l and calcium of 8.6 mg/dl. His was dialyzed on a 2.5 mEq/l calcium bath. He was started on 0.25 mcg of intravenous calcitriol; intravenous epogen and potassium supplements were discontinued. His Sevelamer was changed to 400 mg four times a day with feeds.

Five days after discharge, he developed a hemoglobin level of 6.9 g/dl requiring a 20 ml/kg red blood cell transfusion and was started on maintenance intravenous iron. He was continued on a 2 mEq/L potassium and 2.5 mEq/L calcium dialysate bath. Two days later, during his outpatient HD treatment, he was noted to have hyperkalemia (potassium of 6.6 mEq/L) and was switched to a 1 mEq/L potassium dialysate bath. He had persistent hyperkalemia at his next dialysis visit (potassium level of 6.1 mEq/L) and was started on 10 g of SPS daily. At this time his serum bicarbonate was 28 mEq/L, calcium was 10.6 mg/dL, phosphorus was 7.8 mg/dL, magnesium was 3.8 mg/dL, and intact PTH was 1539 pg/ml (Figure 1(b)). He also had persistent diarrhea. Two days after starting SPS, he had potassium of 4.6 mEq/L and calcium of 7.4 mg/dL. Three days later the patient was noted to be hypokalemic (potassium of 3.4 mEq/L) and he had metabolic alkalosis (serum bicarbonate of 32 mEq/L), hypocalcemia (calcium of 6.3 mg/dL), phosphorus of 10.4 mg/dL, and magnesium 2.9 mg/dL. His heart rate was about 90/min and blood pressure about 110/60 mmHg and he had no tetany, normal deep tendon reflexes, and a negative Chvostek sign. He was admitted and started on calcium carbonate dosed at 200 mg of elemental calcium three times a day (later increased to 270 mg of elemental calcium) and SPS was discontinued. An EKG showed nonspecific ST-T wave abnormalities and normal QT. Telemetry showed appropriate sinus bradycardia overnight without evidence of heart block. His new dialysate contained 1 mEq/L potassium and 4 mEq/L calcium. Calcium levels improved to 8.1 mg/dL, serum bicarbonate of 27 mEq/L, and phosphorus (postdialysis) of 4.4 mg/dL prior to discharge home. Most recently, he has maintained a stable calcium of about 8.8 mg/dL and potassium of 3.7 mEq/L off SPS.

## 3. Discussion

Both the children with CKD and on dialysis were started on SPS for hyperkalemia. Within a week after initiation of the medication, both developed hypocalcemia on routine labs without overt clinical manifestations. The hypocalcemia was rapidly corrected with oral supplementation and discontinuation of SPS.

Potassium exchange resins are used to treat hyperkalemia in CKD frequently. It removes potassium by exchanging sodium ions for potassium ions in the intestine before the resin is excreted. Hypokalemia, hypocalcemia, and even tetany have been described after SPS therapy [1, 2]. A study done by Greenman et al. on patients with congestive heart failure and edema treated with exchange resin therapy showed that four of the twelve patients had abnormally low calcium values after prolonged treatment and three others had only slight decrease in their serum calcium levels [4]. The affinity of ion-exchangers for cations of bivalent elements such as calcium is greater than that for univalent ions such as potassium. Among the univalent ions, the affinity for hydrogen ions exceeds most others [5]. There is a steady loss of calcium in the gut which leads to a risk of gradual demineralization of bone when patients are maintained on cation exchangers for months to years. These studies noted that when patients developed hypocalcemia, the serum calcium levels increased only temporarily with supplementation. The rapid removal of calcium is likely due to deposition in the bones [2, 5]. Our patients however, were noted to have an acute drop in their serum calcium levels within a week of initiation of SPS therapy. Their hypocalcemia responded rapidly to calcium supplementation.

Multiple studies have been done measuring the calcium and potassium content in formulas pretreated with SPS. A retrospective study in Seattle done in 2013 showed, within 72 hours, there was an average 24% decrease in serum potassium after pretreatment of formula or breastmilk with SPS [6]. In 1972, Starbuck showed a decrease in potassium and calcium ion content and an increase in sodium content of milk treated with SPS [7]. A more recent study by Bunchman et al. showed an average drop in calcium levels by 66% in formula pretreated with 1 gm SPS per milliequivalent of potassium in the formula. Human testing in five patients did not show any clinical symptoms of hypocalcemia [8]. Hobbs et al. suggested the use of adult renal formulas that are low in minerals to manage nutrition of hyperkalemic infants with CKD without complications [9].

There are case reports of metabolic alkalosis occurring in patients receiving alkali therapy along with SPS [1, 10]. This occurs because, in the presence of an ion exchange resin, the salt formed by interaction of the antacid with hydrochloric acid in the stomach does not neutralize the bicarbonate in the small bowel. The bicarbonate that is reabsorbed by the gut cannot be excreted in the presence of poor renal function, leading to metabolic alkalosis.

Newer agents, including sodium zirconium cyclosilicate and patiromer have been studied for the use management of chronic hyperkalemia in patients with CKD [3, 11, 12]. Details comparing currently available drugs used for hyperkalemia are shown in Table 1.

Metabolic acidosis of chronic renal failure usually protects patients from overt symptoms of hypocalcemia. Tetany or seizures may occur when acidosis is corrected rapidly without correcting hypocalcemia at the same time.

Severe hypocalcemia can develop after SPS therapy. The metabolic alkalosis in these patients associated with the hypocalcemia put them at increased risk for complications. This report illustrates the necessity of very close monitoring of electrolytes, calcium, and phosphorus balance in pediatric patients on dialysis. Often calcium supplementation is required to maintain normal calcium levels. Newer agents including patiromer and sodium zirconium cyclosilicate are potentially safer medications for management of hyperkalemia in patients with CKD.

## Figures and Tables

**Table 1:** Potassium sparing agents for management of hyperkalemia [3].

	Sodium polystyrene sulfonate (SPS)	Sodium zirconium cyclosilicate	Patiromer
FDA approval	Approved	Still pending	Approved in adults
Mechanism of action	Nonspecific sodium-cation exchange resin	Selective potassium cation binding agent	Calcium based cation exchange resin
Adverse effects	*Colon necrosis*, GI disturbances, hypokalemia, hypomagnesemia, hypocalcemia, metabolic alkalosis	GI disturbances, hypokalemia	GI disturbances, hypokalemia, hypercalcemia, hypomagnesemia
